# Screening for potential targets to reduce stenosis in bioprosthetic heart valves

**DOI:** 10.1038/s41598-021-81340-2

**Published:** 2021-01-28

**Authors:** Rudi Foth, Orr Shomroni, Matthias Sigler, Jürgen Hörer, Julie Cleuziou, Thomas Paul, Katja Eildermann

**Affiliations:** 1 Pediatric Cardiology and Intensive Care Medicine, Georg-August University Göttingen, Robert-Koch Str. 40, 37075 Göttingen, Germany; 2 Department of Developmental Biochemistry, DNA Microarray and Deep-Sequencing Facility, Faculty of Medicine, Georg-August University Göttingen, Justus-von-Liebig-Weg 11, 37077 Göttingen, Germany; 3 Department for Congenital and Pediatric Heart Surgery, German Heart Centre Munich, Lazarettstrasse 36, 80636 Munich, Germany; 4 Division for Congenital and Pediatric Heart Surgery, LMU Klinikum, Marchioninistrasse 15, 81377 Munich, Germany

**Keywords:** Congenital heart defects, Paediatric research, Molecular medicine, Chronic inflammation

## Abstract

Progressive stenosis is one of the main factors that limit the lifetime of bioprosthetic valved conduits. To improve long-term performance we aimed to identify targets that inhibit pannus formation on conduit walls. From 11 explanted, obstructed, RNAlater presevered pulmonary valved conduits, we dissected the thickened conduit wall and the thin leaflet to determine gene expression-profiles using ultra deep sequencing. Differential gene expression between pannus and leaflet provided the dataset that was screened for potential targets. Promising target candidates were immunohistologically stained to see protein abundance and the expressing cell type(s). While immunostainings for DDR2 and FGFR2 remained inconclusive, EGFR, ErbB4 and FLT4 were specifically expressed in a subset of tissue macrophages, a cell type known to regulate the initiation, maintenance, and resolution of tissue repair. Taken toghether, our data suggest EGFR, ErbB4 and FLT4 as potential target candidates to limit pannus formation in bioprosthestic replacement valves.

## Introduction

Reconstruction of the right ventricle to pulmonary artery continuity with homografts and xenografts is a procedure routinely used in patients with certain types of congenital heart defect. Such bioartificial valves have a limited life span ranging between 10 and 15 years^1–4^. After that time, most conduits display severe stenosis leading to functional impairment, insufficient blood supply of the lungs and pressure overload of the right ventricle, requiring surgery^5^. Balloon dilatation and the implantation of a stent mounted valve can delay reintervention for a period of time. Yet, on some point most failing valved conduits need to be replaced surgically using cardio-pulmonary bypass. In a lifetime, a single patient may undergo multiple surgical procedures, thereby accumulating side effects such as surgical risks, scar formation and tissue reactions. Preventing the formation of progressive conduit stenosis could avoid or at least postpone reintervention, reduce the number of hospital admissions and thereby significantly lowering the risk for the patient.

The introduction of an implant into the cardiovascular system triggers a defined series of events, starting with an acute inflammation that is marked by infiltration of immune cells and followed by the formation of granulation-tissue. This mechanism is part of the innate immune system and inter alia aims to convey out the foreign material from the body. When this is unsuccessfull, the inflammation becomes chronic, presenting persistent granulation tissue and the formation of a fibrous capsule that shields the implant from the organism.

Although all parts of the implant are exposed to the same recipient immune system, the magnitude of pannus formation differs. Conduit stenosis develops mainly in areas that alter the flow characteristics of the bloodstream such as a simple folding of the graft-material, thrombus formation, surgical sutures inside the conduit lumen or an offset between the conduit and the native vessel^6^. Detecting the upstream regulators that lead to pannus formation and enable medical intervention to inhibit this process was the aim of the present study.

## Results

### Sample collection

**Figure 1:**
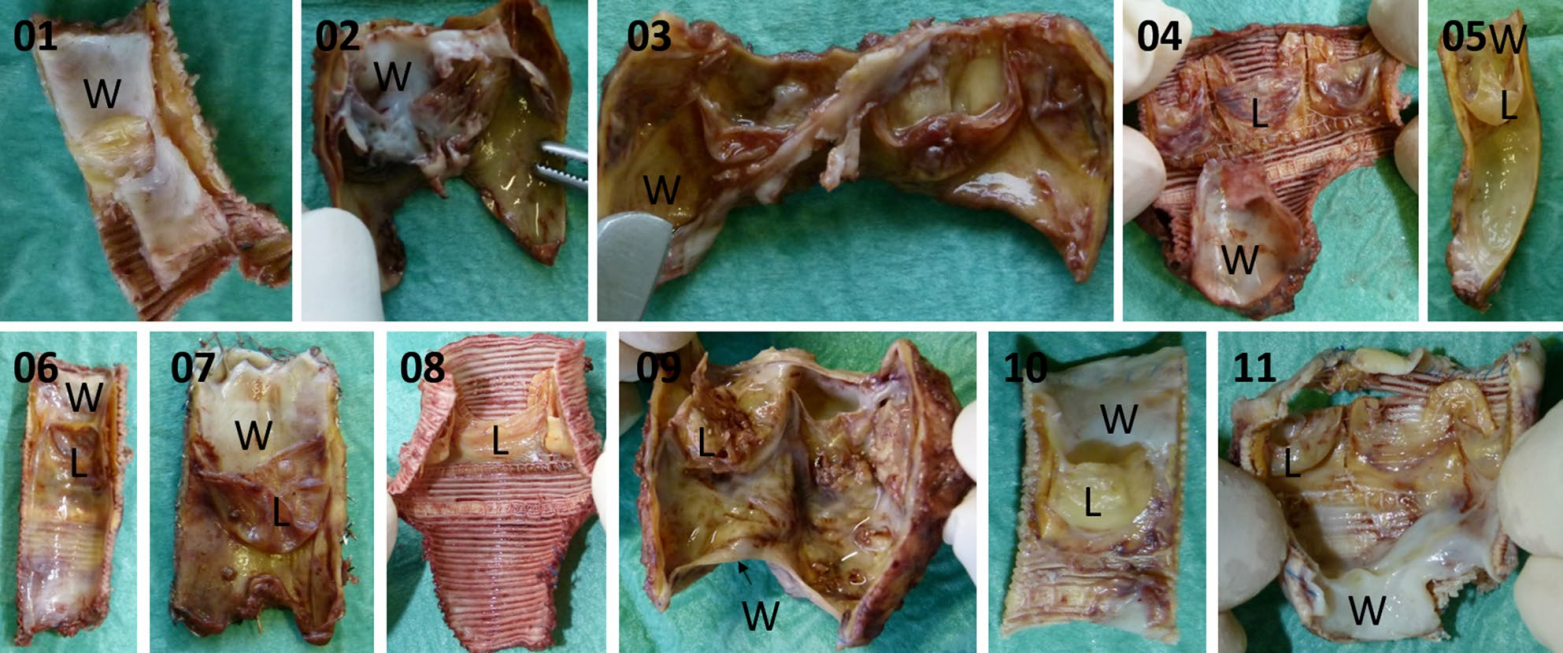
Photographs of all Conduits that were used for NGS. Pannus is recognized as white meterial, graft material of contegra-wall and the leaflets appear yellow / beige and gore-tex material is recognized as wavy fabric. The labels W (thickened wall) and L (leaflet) point out the areas were material was isolated for RNA isolation.

For RNA isolation and sequencing, we collected 11 explanted, dysfunctional conduits. RNA from pannus was obtained from 10 out of 11 conduit walls because one specimen was not obstructed. Leaflet RNA was obtained from 8 out of 11 specimen. Two leaflets were overgrown with fibrous tissue and did not meet our criteria and one leaflet did not yield enough RNA for sequencing. Detailed information on each specimen is listed in Table 1. Photographs of each specimen including an assignment of the areas that were taken for RNA isolation can be viewed in Fig. 1. RNAlater preservation interfered with our histological stainings and the cutting and grinding technique that was nesseccary to section the specimen yielded in a limited number of stainable slides. We therefore used a second set of obstructed conduits for histological analysis of potential target genes.

**Table 1:** Patient characteristics of all analyzed conduits.

Patient	Gender	Diagnosis	Implant	Age at implantation	Duration of implantation	Reason for explantation	Methods applied
NGS 1	Male	PV Agenesis	Hancock	2 m	3 y 5 m	Stenosis	W
NGS 2	female	TAC	Contegra	3 m	2 m	Insufficiency	W
NGS 3	Female	Hemitruncus, VSD, PS, PFO	Contegra	2 m	6 m	Insufficiency	W
NGS 4	Male	TOF, PA	Hancock	19 y 4 m	4 y 8 m	Stenosis	L, W
NGS 5	Male	DOLV, MGA, PS	Contegra	2 y 7 m	11 y 11 m	Stenosis	L, W
NGS 6	Male	TAC, ASD	Hancock	2 m	5 y 0 m	Stenosis, Insufficiency	L, W
NGS 7	Female	VSD, PA	Melody in Homograft	18 y 5 m	4 y 8 m	Stenosis	L, W
NGS 8	Male	TAC, ASD	Hancock	5 y 2 m	2 m	Endocarditis	L
NGS 9	Male	AS, Ross-Procedure	Contegra	1 y 10 m	9 y 1 m	Stenosis, Endocarditis	L, W
NGS 10	Male	d-TGA, VSD	Hancock	5 y 10 m	14 y 10 m	Stenosis	L, W
NGS 11	Female	DORV, VSD, PA	Hancock	11 m	6 y 7 m	Stenosis	L, W
Histo 1	Male	DORV, TOF	Hancock	5 m	9 m	Stenosis	Histology
Histo 2	Male	PA, VSD	Hancock	9 m	9 m	Stenosis	Histology
Histo 3	Female	DORV, TOF	Hancock	11 m	9 m	Stenosis	Histology
Histo 4	Male	DORV, TGA	Hancock	3 m	1 y 2 m	Stenosis	Histology
Histo 5	Female	DORV	Hancock	1 y 3 m	1 y 5 m	Stenosis	Histology
Histo 6	Male	PA, VSD	Hancock	1 y 2 m	3 y 1 m	Stenosis	Histology
Histo 7	Male	TOF, PS	Hancock	7 m	5 y 8 m	Stenosis	Histology
Histo 8	Male	TGA, VSD	Hancock	6 y 10 m	15 y 6 m	Stenosis	Histology
Histo 9	Female	TOF	Contegra	1 y 5 m	1 y 2 m	Stenosis	Histology
Histo 10	Male	PA, VSD	Matrix P	1 m	10 m	Stenosis	Histology
Histo 11	Male	Dysplastic AV	Homograft	41 y 1 m	9 y 5 m	Stenosis	Histology

AS, aortic stenosis; ASD, atrial septal defect; AV, aortic valve; d-TGA- dextro-transposition of the great arteries; DOLV, double outlet left ventricle; DORV, double outlet right ventricle; L, leaflet; MGA, malposition great arteries; PA, pulmonary atresia; PFO, patent foramen ovale; PS, pulmonary stenosis; PV, pulmonary valve; TAC, truncus arteriosus communis; TGA, transposition of the great arteries; TOF, tetralogy of Fallot; VSD, ventricular septal defect; W, wall; NGS, next generation sequencing.

### Next generation sequencing and differential gene expression

**Figure 2:**
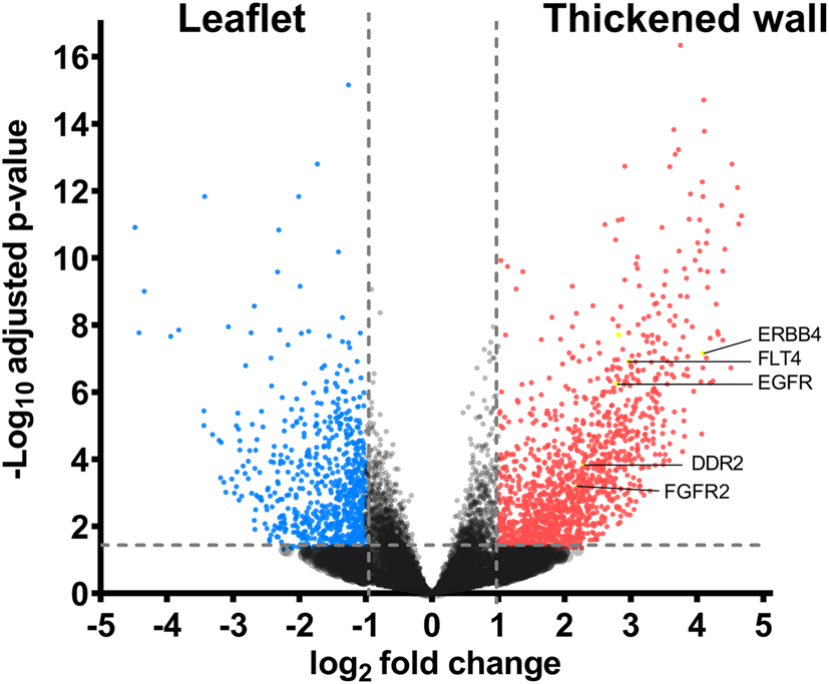
Vulcan blot showing deregulated genes in the conduit leaflets (left, n = 8) and thickened conduit wall (right, n = 10). Each red dot represents a single gene. Deregulated tyrosine kinases that were selected for further analysis are labelled.

NGS raw data are available in the Gene Expression Omnibus from NCBI at https://www.ncbi.nlm.nih.gov/geo/, and can be accessed with GSE150288. Differential gene expression analysis revealed an upregulation of 1023 genes in the leaflet and 1898 genes in the pannus (see Fig. 2 and supplement 1). We used the reactome pathway knowledgebase^7^ to obtain a more global view on the deregulated genes. Within the leaflets, we found an enrichment of genes contributing to (1) neutrophil degranulation as part of the innate immune system, (2) hemostasis such as fibrin clot formation, platelet activation and platelet adhesion to collagen and 3) collagen degradation. Thickened conduit walls were enriched for (1) extracellular matrix related genes such as ECM-proteoglycans, collagen, elastin and laminin and (2) genes related to smooth muscle contraction. Most important for this project was the finding of (3) the enriched reactome pathway “signaling by receptor tyrosine kinases” in the thickened wall, that involves proteins with the potential for medical intervention. 12 transmembrane receptor tyrosin kinases (RTK) were upregulated with a log2 FC > 2 in the pannus (see Table 2 and supplement 2). From this group of proteins EGFR, FGFR2, DDR2, ErbB4 and FLT4 were selected for immunohistological analysis in methylmethacrylat embedded ground cuts of explanted obstructed conduits.

**Table 2:** Receptor tyrosin kinases upregulated in pannus of conduit wall (log2 FC > 2 and padj < 0.05).

Gene name	log2 fold change	P adjusted
ERBB4	4.09	6.94E-08
PDGFRA	3.00	1.53E-07
PDGFRB	3.01	2.93E-07
FLT4	2.97	1.20E-07
NTRK3	2.82	1.97E-08
ROR2	2.81	1.05E-04
EGFR	2.79	5.63E-07
ROR1	2.37	1.90E-05
DDR2	2.28	1.39E-04
EPHA3	2.25	8.68E-03
PDGFRL	2.22	3.47E-03
FGFR2	2.19	6.47E-04

### Immunohistochemistry

The dominating tissue type on the luminal side of the conduit-wall was fibrous, with a high proportion of extracellular matrix and the typical spindle shaped fibroblasts. However, it was striking that sections with very thick fibrous neo-tissue also exhibited more granulation tissue, with low to no extracellular matrix, many immune cells such as plasma cells, lymphocytes and macrophages and capillaries.

**Figure 3:**
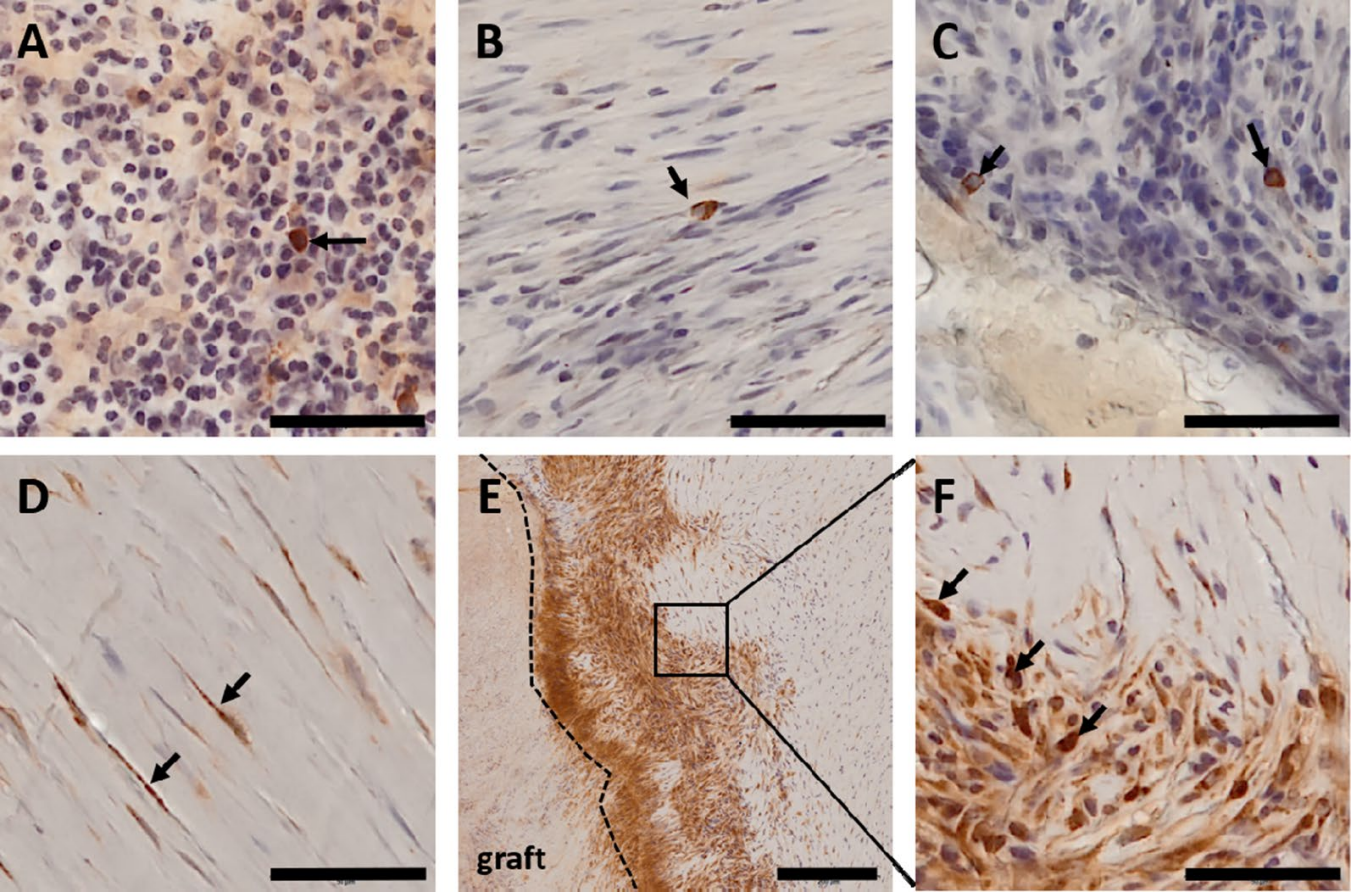
Immunohistochemical staining of candidate genes (brown). Nuclei were stained with hematoxylin and appear in blue. All pictures show neotissue in the thickened area of the conduit wall. **(A)** Black arrow points to **EGFR** positive macrophage in granular tissue. **(B)** Black arrow points to **ErbB4** positive macrophage in transition area between granular and fibrous tissue. **(C)** Black arrows point to **ErbB4** positive macrophages in granular tissue. **(D)** Black arrows point to **FLT4** positive, spindle shaped fibroblasts in fibrous tissue. **(E)** Overview picture of **FLT4** staining. Graft material is located on the left side of the picture. A dotted line marks the border between graft and neo tissue. Neo tissue close to the graft was mostly granular, becoming more fibrous towards the lumen (right side in the picture). Here, granular tissue was generally intensively stained, whereas in fibrous tissue, staining was restricted to the fibroblasts. Square indicates the area that is zoomed in Fig. 10.1038/s41598-021-81340-2F. **(F)** Close up of square in Fig. 10.1038/s41598-021-81340-2E. Black arrows point to more intensively stained FLT4 positive macrophages. Scale bars: 50 µm; scale bar Figure E: 200 µm. Stainings were perfomed on n = 11 explanted conduits.

The immunostainings for DDR2 and FGFR2 remained largely inconclusive. While EGFR had a relatively high background staining throughout the tissue, it was more abundant in the cytoplasm of some plasma cells. Clearest signals were detected on the membranes of a small macrophage-subgroup within granulation tissue close to foreign material (Fig. 3A). ErbB4 was also detected on the membranes of a subset of tissue macrophages within granulation tissue (Fig. 10.1038/s41598-021-81340-2B,C). FLT4 strongly stained the complete granulation tissue near the graft material (Fig. 3D–F). Though, a few macrophages stand out exhibiting intense surface staining. Within the fibrous tissue close to the luminal surface, some very thin, spindle shaped fibroblasts embedded in extracellular matrix stained positive for FLT4. Macrophages were identified by morphology.

### Impact of implant type, duration of implantation and age at implantation

To see if (A) type of material (B) duration of implantation or C) age at implantation had an influence on the expression levels of FLT4, ErbB4 and EGFR, we divided the specimen into the following groups: (A) bovine (n = 5) and porcine (n = 6),B) < 1 year (n = 3), 1–9 years (n = 5) and 10–19 years (n = 3), (C) < 1 year (n = 1), 1–9 years (n = 4), 10–19 (n = 2) and 20–29 years (n = 3). Although we detected differentially expressed genes between groups larger than or equal to n = 3, there were no relevant differences for the RTKs upregulated in pannus. Supplement 2 provides the individual expression levels for all RTKs that were upregulated > 2 log2 FC.

## Discussion

In this study, we compared the gene expression profile of pannus to the gene expression profile of thin leaflets to screen for genes that regulate the different tissue reactions of these two different areas of the implant. Global analysis of deregulated genes provided a proof of principle for our method. The reactome pathways knowledgebase showed an enrichment of acute processes in the leaflets. This parallels our experience from histological evaluation of explanted, dysfunctional conduits where we frequently see fibrin condensation, platelet adherence and adhering neutrophils. These events most likely relate to leaflet degradation, another big issue with bioprosthetic replacement valves. In the conduit wall, reactome pathways indicate the typical scar-like histology with a high amounts of extracellular matrix, matrix producing fibroblasts and myofibroblasts that we see in histology^8^. The overall higher number of deregulated genes in the pannus is most likely due to a wider variety of cell types.

Concerning potential targets, we found an enrichment of “signaling by receptor tyrosine kinases” in the thickened material. In our analysis we focused on these RTKs. RTKs exhibit an extracellular binding domain that upon binding transfers information into the cell thereby regulating cell growth, proliferation, migration and survival^9^. Many diseases and cancer types are attributed to dysfunctional RTK signalling^10^. RTK inhibitors are therefore promising candidates for the regulation of diseases. In cancer therapy, RTK inhibitors are already widely applied^9^. An RTK inhibitor was studied for the treatment of pulmonary vein stenosis^11^, as Riedlinger et al*.* had described vascular endothelial growth factor receptor (VEGFR) enriched in the thickened proportion of the stenosed vessels^12^. Here, we used a similar approach and hypothesized that the RTKs in our specimen were involved in the formation of conduit stenosis, e.g. by regulating excessive cell proliferation, excessive formation of granulation tissue or excessive production of ECM.

The epidermal growth factor receptor (EGFR) was significantly upregulated by 2.8 fold in thickened conduit walls compared to the leaflets. Our histological findings indicate an impact of EGFR in macrophages. Here EGFR-signaling plays a substantial role in macrophage activation^13, 14^. Hoyer et al*.* showed that EGFR-deficient macrophages have an impaired Th1 and Th17 adaptive immune response to *H. pylori*, leading to decreased chronic inflammation in infected mice^13^. A decrease in macrophage activation and a decrease in neutrophil and T-cell infiltration in myeloid-EGFR knockout-mice has been shown in a study by Hardbower et al*.*^14^. They demonstrated a combined effect of restrained M1 and M2 macrophage activation resulting in a decreased production of the pro-angiogenic factors CXCL1 and VEGF, and consequently to a reduced number of CD31 positive blood vessels. These data imply that presence of EGFR positive macrophages may reflect an activated macrophage state accounting for an unresolved immune response. Successful blockage of EGFR reduces chronic inflammation and fibrocyte recruitment to stop successive capsule expansion^15^. Blocking EGFR, either via covering the kinase domain to stop EGFR activity or by covering the extracellular domain using monoclonal antibodies to inhibit receptor dimerization is an established treatment for different types of cancers^16^ and could be adapted to treat conduit stenosis.

ErbB4, the second epidermal growth factor receptor in our dataset, is known to regulate proliferation and migration. Immunohistology revealed a clear membrane staining on macrophages in granular neotissue. Previous research on ErbB4 in macrophages described a role in the stimulation of pro-inflammatory macrophage apoptosis^17^. Efficient clearance of pro-inflammatory macrophages from tissue after the resolution of an activating stimulus is critical. Any errors during this process potentially lead to prolonged or chronic inflammation and eventually the deposition of excessive fibrous tissue^18, 19^. The binding of neuregulin 4 (NRG4) appears to be mandatory to activate the apoptosis pathway through ErbB4 in macrophages^17^. As our data did not reveal any evidence for NRG4, local application of this factor may activate apoptosis in ErbB4 positive macrophages, thereby resolving the state of chronic inflammation.

Fms related receptor tyrosine kinase 4 (FLT4), also known as vascular endothelial growth factor receptor 3 (VEGFR-3) is an important regulator of adult lymphangiogenesis^20^ in cancer^21, 22^ and wound healing^23^. FLT4 expressing macrophages are known to induce lymphangiogenesis in cancer^24^ while blocking of FLT4 suppresses angiogenic sprouting and vascular network formation^25^. As demonstrated previously, FLT4 can be bypassed by adding soluble FLT4. This approach has already been shown to suppress lymphangiogenesis and lymphatic metastasis in bladder cancer^26^.

Our data together with the current literature indicates a role of macrophages in the development of conduit stenosis. Macrophages are phenotypically extremely flexible which enables them to regulate initiation, maintenance, and resolution of tissue repair. Any disturbances in macrophage function can lead to uncontrolled production of inflammatory mediators and growth factors or deficient generation of anti-inflammatory macrophages. Failed communication between macrophages and other cell types may result in a state of chronic inflammation with development of pathological fibrosis^27^. Influencing the macrophage phenotype towards pro-inflammatory macrophage apoptosis via ErbB4 or reducing the amount of activated macrophages via EGFR can potentially lead to the resolution of the chronic inflammation that is caused by the implant. Reduction of endothelial sprouting reduces the networking of neotissue thereby potentially liming the extent of fibrous tissue production that causes the obstruction.

Due to the descriptive nature of evaluating human material, our study has limitations.

We aimed to find medical targets to treat conduit stenosis across all implant-types and included all cases of conduit stenosis that we were able to collect in a given timeframe. However, we were aware that immune reactions might differ, depending on A) type of material B) duration of implantation and C) age at implantation. As described in "Impact of implant type, duration of implantation and age at implantation" section. None of these variables showed significant impact on gene expression of EGFR, ErbB4 or FLT4 or the other RTKs in our NGS dataset.

Methological difficulties hindered the use of the same set of explanted conduits for NGS and Immunohistology. While RNA-Sequencing was applied to find differentially expressed genes between the conduit leaflet and the thickened conduit wall. Histology was not applied to confirm these findings, but to detect the location of the gene-products within thickened conduit wall and closer define the expressing cell types. The aim of histology was not quantitative, but qualitative.

The cutting and grinding method results in one staining every 100 – 150 µm. As this competes with a large number of different stainings, the identification of the marker-positive cells as macrophages was based on morphology only. Also, this limitation hinders the evaluation of a greater amount of genes from our list of differentially expressed genes. However, our data set might provide more potential targets than receptor tyrosine kinases.

Children in which the valved conduits are initially implanted are predominantly less than 2 years old and in a developmental stage that is marked by excessive cell proliferation and growth. Although our strategies aim to reduce pro-inflammatory macrophages in granular tissue, the influences of potential medications on the proliferation of somatic cells must be examined. Local application e.g. via immobilization on the implants surface is desirable. This would shield the medication from global distribution and lower potential side effets. However this strategy needs further extensive testing and escaping vehicles must be studied and keept to a minimum.

## Materials and methods

### Patient cohort

Clinical data of the patients were obtained from medical records. The study was approved by an institutional review committee (Ethics committee of the medical faculty, Georg-August-Univeristät Göttingen). Informed written consent was given prior to inclusion of subjects in the study. Underaged patients were informed in an age-approriate manner and informed witten consent was given by the parents. Data were processed and documented according to the Declaration of Helsinki.

We used 11 RNAlater preserved explanted valved conduits for next generation sequencing (NGS). RNAlater preservation was mandatory for RNA sequencing but impeded immunohistological staining. Therefore, a different set of 11 Conduits was used for immunohistology. These were fixed in formalin after explantation. Table 1 provides all relevant details for each specimen used in this study.

### Sample preparation for next generation sequencing (NGS)

Conduits for NGS were explanted and immediately transferred to RNAlater (Sigma Aldrich) to inhibit RNA degradation. Explants were then dissected to isolate the thickened material of the conduit wall and one of the three valve-leaflets for RNA isolation (as indicated in Fig. 1). RNA isolation was performed in an RNase free area using the TRIzol reagent (ThermoFisher, 15596026) according to manufacturer’s recommendations. Briefly, small tissue-pieces were frozen in liquid nitrogen and powdered using the TissueLyser (Qiagen). Resulting tissue-powder was lysed in TRIzol reagent and RNA was precipitated using a phenol / chloroform extraction. DNase treatment was applied and RNA quality was evaluated using the NanoDrop (ThermoFisher) and a fragment analyzer. RIN values are listed in Supplement 3. RNA-seq libraries were prepared using 500 ng total RNA of a non-stranded RNA Seq, massively-parallel mRNA sequencing approach from NEB.

### Raw read & quality check

Sequencing was perfomed using the Illumina HiSeq 2500. Sequence images were transformed with Illumina software BaseCaller to BCL files, which was demultiplexed to fastq files using CASAVA-1.8.2. The sequencing quality was asserted using FastQC^28^ (version 0.11.5).

### Mapping & normalization

Samples were aligned to the reference genome *Homo sapiens* (hg38 version 97, https://www.ensembl.org/Homo_sapiens/Info/Index) using the STAR aligner^29^ (version 2.5.2a) allowing for 2 mismatches within 50 bases. Subsequently, reads were quantified for all hg38 version 97 genes in each sample using featureCounts^30^ (version 1.5.0-p1). Read counts were analyzed in the R/Bioconductor environment (version 3.6.2, www.bioconductor.org) using the DESeq2^31^ package version 1.24.0. In DESeq2, the p-values attained by the Wald test are corrected for multiple testing using the Benjamini and Hochberg method. Genes were annotated using the *Homo sapiens* GTF file used to quantify reads. Deregulated genes were those with adjusted *p* value ≤ 0.05 and an absolute log2 fold-change ≥ 1.

### Immunohistochemistry

Conduits for immunohistology had been explanted and immediately transferred to formalin to preserve tissue integrity. Surgically removed implants often contain proportions of metal or plastics impeding standard microtome sectioning. Therefore, explanted conduits were embedded in methylmethacrylate (Technovit 9100, Kulzer & Co, Wehrheim, Germany). Following curing, resin blocks were fixed to a glass slide using a silicon-based adhesive (Elastosil E41, Wacker Chemie GmbH, München, Germany). Using a diamond saw (300 CP, Exakt GmbH, Norderstedt, Germany) and a rotational grinding machine (400 CS, Exakt GmbH, Norderstedt, Germany), samples were trimmed down to a thickness of 5–30 µm^32, 33^. Immunohistochemistry was performed as previously described^33^. Briefly, slides were deplastinized by incubation in a series of xylene, 2-methoxyethylacetat, acetone and water, followed by specific antigen retrieval that was dependent on the antibody (see Table 3). After blocking endogenous peroxidase, the first antibody was incubated over night at room temperature. After washing, the secondary antibody was applied followed by polymer signal amplification. Further details about the antibodies, dilutions and necessary pre-treatments are listed in Table 3. All stained sections were covered and scanned using the Dotslide system (Olympus).

**Table 3:** Antibodies used in this study, including exact antibody information, dilutions, pre-treatments, and detection system used.

Antigen	Antibody	Company	Dilution	Pre-treatment	Secondary antibody	Detection system
EGFR	Monoclonal Mouse Anti Human EGFR	DAKO M3563	1:500	Prot. K	Swine Anti Rabbit Immunglobulin DAKO P0399	ZytoChem Plus (HRP) Polymer anti-Rabbit ZUC032-006
ErbB4	Monoclonal Mouse IgG 2b Anti ErbB4	Thermo Fisher MA1-861	1:100	TR 6,1	Swine Anti Rabbit Immunglobulin DAKO P0399	
FLT4	Monoclonal Rabbit Anti Human FLT4	Spring E3870	1:300	TR 6,1	Rabbit Anti Mouse Immunglobulin DAKO P0260	ZytoChem Plus (HRP) One-Step Polymer anti-Mouse/Rabbit ZUC053-006
FGFR2	Monoclonal Rabbit Anti Human	Spring M5730	1:300	TR 6,1	Rabbit Anti Mouse Immunglobulin DAKO P0260	ZytoChem Plus (HRP) One-Step Polymer anti-Mouse/Rabbit ZUC053-006
DDR2	Monoclonal Mouse Anti IgG2a	Thermo Fisher MA5-15356	1:200	TR 6,1	Rabbit Anti Mouse Immunglobulin DAKO P0260	

## Supplementary Information

Supplementary Table 1

Supplementary Table 2

Supplementary Table 3

## Abbreviations

CD31: Cluster of differentiation 31
CXCL1: Chemokine (C-X-C motif) ligand 1
DDR2: Discoidin domain-containing receptor 2
ECM: Extracellular matrix
EGFR: Epidermal growth factor receptor
ErbB4: Receptor tyrosine-protein kinase erbB-4
FGFR2: Fibroblast growth factor receptor 2
FLT4: Fms-related tyrosine kinase 4
NGS: Next generation sequencing
NRG4: Neuregulin 4
RNA: Ribonucleic acid
RTK: Receptor tyrosine kinases
VEGFR: Vascular endothelial growth factor receptor
